# Pulmonary Embolism Presenting as Abdominal Pain and Asystole

**DOI:** 10.7759/cureus.9123

**Published:** 2020-07-10

**Authors:** Abjad Al Busaidi, Mustafa Al Balushi, Nasser Al Busaidi

**Affiliations:** 1 Internal Medicine, Oman Medical Specialty Board, Muscat, OMN; 2 Radiation Oncology, University of Alberta, Edmonton, CAN; 3 Chest Medicine, Royal Hospital, Muscat, OMN

**Keywords:** pulmonary embolism, abdominal pain, asystole

## Abstract

Pulmonary embolism (PE) is a life-threatening condition that mandates prompt identification and management. The protean and atypical symptomatology of PE can mislead the physician and pose a diagnostic dilemma. Abdominal pain is one such rare symptom that is not commonly encountered in the clinical setting. With the limited availability of literature describing abdominal pain as a symptom of this acute disease, it is pivotal that healthcare workers are aware of this presentation. Herewith, we report a 36-year-old man with no co-morbidities who presented with abdominal pain and subsequent cardiac arrest. He was diagnosed and managed in the emergency department and made a complete recovery.

## Introduction

Pulmonary embolism (PE) is a common condition in clinical practice. It has been found that the acute onset of dyspnea is the most common presenting symptom [1]. Other common symptoms include pleuritic chest pain, cough, orthopnea, symptoms of deep vein thrombosis, wheezing, and hemoptysis. Some uncommon presentations have been documented in the literature [2]. Prompt identification and management of this treatable disease is achievable with recognition of the typical patterns of presentation. However, a vigilant clinician should be aware of the unusual clinical presentations of this disease. The mortality rate of untreated or missed PE is around 30% [3]. Herein, we present a case of a 36-year-old male with no co-morbidities who presented to the emergency department with acute abdominal pain and was eventually diagnosed and managed as PE.

## Case presentation

**Figure 1:**
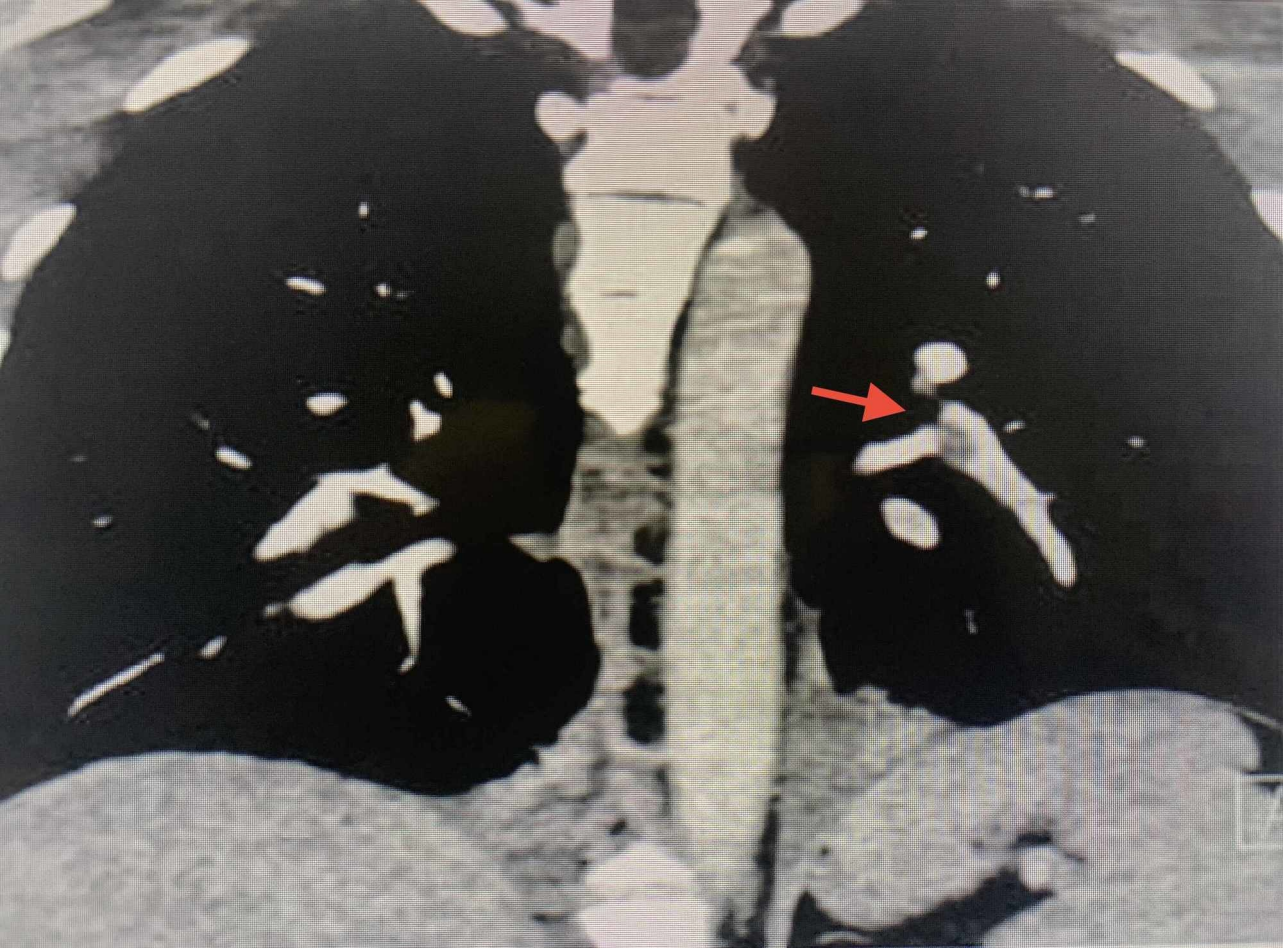
Coronal slice of the computed tomography pulmonary angiography showing segmental and subsegmental filling defects in the left pulmonary artery.

**Figure 2:**
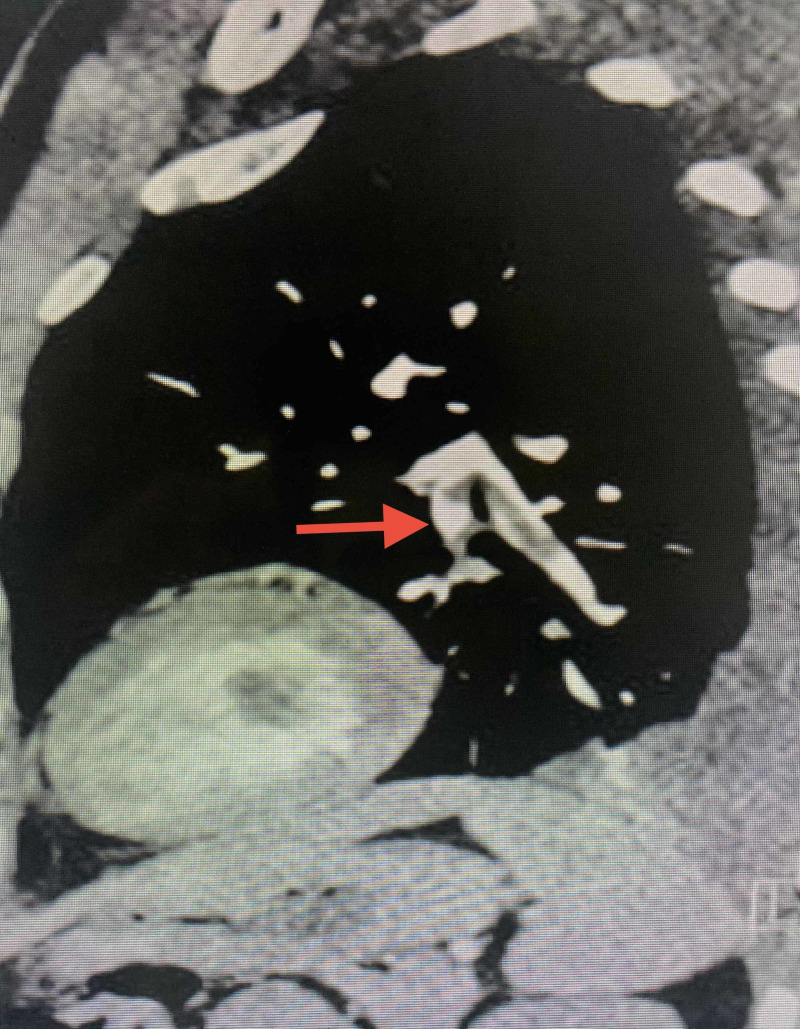
Sagittal slice of the computed tomography pulmonary angiography showing segmental and subsegmental filling defects in the left pulmonary artery.

A 36-year-old male with no known co-morbid conditions presented to the emergency department with acute onset of diffuse excruciating abdominal pain. The patient was attended immediately and was transferred to the resuscitation room of the triage section. He was sweating profusely and he collapsed after transfer to the triage room. He became cyanosed with a raised jugular venous pressure (JVP) compounded with cardiopulmonary arrest. Cardiopulmonary resuscitation according to advanced cardiac life support (ACLS) protocol was initiated, and the patient was found to have asystole. He was revived after two minutes of resuscitation. Shortly afterward, he sustained a second cardiac arrest and the trace showed pulseless electrical activity, and resuscitation was resumed. Arterial blood gas was performed and revealed severe mixed acidosis. His pH was 6.77, pCO_2_ 77 mmHg, pO_2_ 64.7 mmHg, bicarbonate 7 mmol/L, and base excess -23.8 mmol/L. His electrolytes were within normal limits. Bedside echocardiography was obtained, which showed a right ventricular strain and an ejection fraction of 60%. A diagnosis of PE was suspected to be the offender. During resuscitation, he was intubated and ventilated, and a detailed history was taken from the patient’s partner. It was found that he underwent bilateral arthroscopy a day prior to presentation and was discharged uneventfully. Subsequent to this significant history, a diagnosis of PE was provisionally made. A computed tomography pulmonary angiography (CTPA) was contemplated to confirm the diagnosis. However, due to his hemodynamic instability, this was deferred. A decision was made for thrombolysis during resuscitation, so he was thrombolyzed with two doses of retaplase half an hour apart. Shortly after thrombolysis with retaplase, his cyanosis and JVP improved drastically and he was revived. His vital signs started to improve dramatically and he continued to improve thereafter. Repeat echocardiography after thrombolysis showed mild improvement in the right ventricular strain. Subsequently, he was transferred to the intensive care unit for close monitoring and further anticoagulation as per protocol. A CTPA was done, which showed evidence of filling defects within the left lower lobe, segmental and subsegmental arteries as well as in the right lower lobe basal lateral segmental artery in keeping with pulmonary emboli (Figures 1, 2). After spending five days in the ICU, he was deemed stable enough to be transferred to a regular ward. He was discharged from the hospital after a total of 10 days in the hospital in good condition. In subsequent follow-up appointments, the patient was found to be completely healthy with no long-term sequelae. 

## Discussion

PE is a life-threatening condition that warrants management in a timely fashion. This condition can easily be missed due to its wide range of clinical presentations [4]. However, there is a constellation of signs and symptoms that often lead the clinician to clinch the diagnosis [1]. It has been found, however, that there is a population of patients who present atypically which makes prompt management a challenge [2]. Those rare presentations include syncope, cardiac arrhythmias, and their associated symptoms and abdominal pain [2,5]. Amesquita et al. reported flank pain in a case series [6]. Carrascosa et al. studied elderly patients who presented with delirium who were subsequently diagnosed with PE [7]. Seizures, fever, cough, and wheezing were all reported in the literature [8]. Since our patient in this case report presented with abdominal pain, it is worthwhile noting that only 6.7% of patients who are eventually diagnosed with PE present this way [9].

The underlying pathogenesis of abdominal pain in PE is not adequately understood. However, it is hypothesized that there are various mechanisms that cause abdominal pain including diaphragmatic irritation, distension of the liver capsule (Glisson's capsule), and hepatic congestion due to a right heart strain [1,10,11]. It has been hypothesized that what usually happens in massive PE that there is a large clot obstructing the pulmonary trunk causing a sudden and rapid increase in pulmonary artery pressure leading to an acute right ventricular failure (RVF) [12].

RVF will cause direct backpressure on hepatic veins and sinusoids leading to passive congestion of the liver causing severe and intolerable pain [13]. A different pathophysiological explanation is that the sensory nerve endings or emboli in the vasculature of the mesentery may be contributing factors [1,10,11].

It is worth mentioning that the mortality rate goes up to 58.3% when patients with massive PE present hemodynamically compromised in comparison to those who present with stable vitals with a percentage of 15.1% [13,14].

There are no clear guidelines in regard to the use of thrombolytic agents in massive PE. However, there is literature that supports the use of thrombolysis in hemodynamically unstable patients since the survival rate is 19% compared to 9% for those who have received only unfractionated heparin [15].

The initial presentation of the patient would have led to a different diagnosis. However, a high index of suspicion was maintained throughout the resuscitation period due to the sudden cardiac arrest and the strongly suggestive history. Hence, the patient was managed along the lines of PE, which resulted in his recovery from the cardiovascular collapse.

## Conclusions

Acute abdominal pain is a rare presentation of PE. Therefore, a high index of suspicion should be maintained when a patient presents with abdominal pain and a history suggestive of PE. A prompt diagnosis and management can save the patient’s life and reduce the associated morbidity. A physician should be vigilant of different causes when a previously healthy adult presents with unexpected cardiac arrest.
